# N-Lactoyl amino acids as metabolic biomarkers differentiating low and high exercise response

**DOI:** 10.5114/biolsport.2025.145912

**Published:** 2024-12-19

**Authors:** Maha Sellami, Khaled Naja, Shamma Almuraikhy, Najeha Anwardeen, Rinat I. Sultanov, Eduard V. Generozov, Ildus I. Ahmetov, Mohamed A. Elrayess

**Affiliations:** 1 Physical Education Department (PE), College of Education, Qatar University, Doha, Qatar; 2 Biomedical Research Center, Qatar University, Doha P.O Box 2713, Qatar; 3 Department of Molecular Biology and Genetics, Federal Research and Clinical Center of Physical-Chemical Medicine of Federal Medical Biological Agency, Moscow, Russia; 4 Laboratory of Genetics of Aging and Longevity, Kazan State Medical University, Kazan, Russia; 5 Research Institute for Sport and Exercise Sciences, Liverpool John Moores University, Liverpool, UK; 6 College of Medicine, QU Health, Qatar University, Doha P.O Box 2713, Qatar

**Keywords:** N-lactoyl amino acids, Phe-Lac, Biomarkers, Metabolomics, Exercise response, 6-minutes walking test

## Abstract

Aerobic physical exercise has significant benefits for cardiovascular health; however, some individuals experience no benefit or even adverse effects. One reason for poor tolerance to aerobic exercise may be a low percentage of slow-twitch (oxidative) muscle fibers. This study aims to identify the metabolic signatures associated with low and high response to exercise by comparing the metabolic profiles of participants categorized according to their improvement of the 6-minute walking distance. In this study, pre- and postexercise intervention measurements of the 6-minute walking distance were conducted in forty-three lean and overweight young women, followed by non-targeted metabolomics analysis of 1039 known metabolites. An independent validation cohort comprising 791 individuals from the GTEx project was used to assess the gene expression of selected targets. The results indicated that a low improvement in the 6-minute walking distance (Δ 6-MWD = 27 meters) was associated with higher serum levels of N-lactoyl amino acid metabolites, particularly the exercise-inducible metabolite N-lactoyl phenylalanine (Lac-Phe) (FDR = 0.016), compared to high responders. Our results were corroborated in an independent validation cohort, which showed that the gene expression of cytosolic nonspecific dipeptidase (*CNDP2*), the enzyme responsible for Lac-Phe synthesis, is negatively associated with the percentage of slow-twitch muscle fibers (*p* < 0.0001). N-lactoyl amino acids may serve as biomarkers for rapid muscle fatigue and low response to exercise, and could be used as metabolic indicators to differentiate exercise response efficacy.

## INTRODUCTION

Aerobic fitness is the capacity to perform prolonged, high-intensity activities, predominantly relying on aerobic metabolism. Physical activities such as mid- and long-distance running/walking are prime examples that demand high levels of aerobic fitness. Aerobic physical exercise has been consistently linked to improved cardiac structure, function, and cardiovascular risk profiles, highlighting its positive impact on cardiovascular health [1–3].

Several studies have found that some individuals experience beneficial effects of exercise on metabolic health, while others show no change or even adverse effects [4, 5]. The mechanisms underlying the variability in exercise responsiveness, as well as potential predictors, remain unclear at present [6]. Measuring exercise effectiveness through pre- and post-training assessments is crucial for quantifying the impact of training initiatives and facilitating necessary adjustments for continuous improvement.

The 6-minute walking test (6-MWT) measures the distance an individual can walk on a flat surface in 6 minutes, which reflects their functional exercise level and aerobic capacity. The 6-MWT is a valuable tool for measuring the variability of functional exercise capacity and response to exercise training in clinical populations. Its simplicity and correlation with real-world activities make it a preferred outcome measure in many exercise intervention studies [7].

Measuring the difference in 6-minute walking distance (Δ6-MWD) before and after exercise can provide valuable insights into the individual’s response to aerobic exercise.

Metabolomics is becoming increasingly important in the field of exercise physiology and sports science. By analyzing metabolic profiles, researchers can gain insights into the complex physiological changes that occur in response to exercise [8].

Our objective is to identify metabolic signatures associated with low and high response to exercise. This could lead to the discovery of novel therapeutic targets to enhance the metabolic benefits of exercise, and improve our understanding of the mechanisms linking metabolism and poor exercise outcomes. In this study, we conducted a controlled exercise intervention in lean and overweight young women followed by non-targeted metabolomics analysis.

## MATERIALS AND METHODS

### Study participants

Forty-three female students from Qatar University, who were not regularly participating in physical activity, took part in this study. Inclusion criteria included a BMI above 20 and below 30 kg/m^2^, and age between 20 and 30 years old. Participants with any cardiovascular condition, type 2 diabetes, muscle degeneration, blood clots, and neurological disorders were excluded. All participants provided a consent form prior to participation. All protocols were approved by Qatar University (QU-IRB 1798-EA/23) as per regulations of the Qatar Ministry of Public Health (MoPH).

The validation cohort includes 791 individuals from the GTEx project which involved 535 males (age 20–79 years) and 256 females (age 20–79 years) of European descent, as previously described [9]. The GTEx study (dbGaP accession number phs000424. vN.pN) was approved by local Ethics Committees, as previously described [9].

### Study design

Participants were engaged in an aerobic training session for 4 to 8 weeks. The training program, adhering to American College of Sports Medicine (ACSM) and American Heart Association (AHA) recommendations [10−13], comprised aerobic exercises with progressive intensity (40–60% of HRmax and 50% of VO_2_ peak initially, progressing to 60–70% by the 4^th^ or 8^th^ week). All participants were trained three days per week for 50 minutes per session. The Metabolic Equivalent of Task (MET) values were adjusted based on IPAQ responses to quantify daily activities. MET was utilized for intensity and energy expenditure, expressed similarly for individuals of different weights. An assessment of 6-MWT was done both before and after the training intervention. The test was standardized for all participants and specific instructions were given before and during the test to ensure consistency and accuracy in the results. Each participant received a detailed description of the methodology before providing signed informed permission. As per the ATS statement [14], the 6-MWT was carried out with close observation, motivational words from the researcher, and tracking of dyspnea, SaO_2_, and muscular exhaustion. All participants should have eaten at least two hours before the test, worn proper shoes, and worn comfortable attire on the day of the test. Study participants were not engaged in regular physical training before experimentations, hence, a 6-MWT would be a valuable tool to evaluate their submaximal exertion and endurance. Recent literature has shown that the assessment of aerobic capacity is crucial in both clinical and fitness settings for populations that may not engage in regular exercise, and emerging data for young healthy adults suggest that the 6-MWT is a valuable tool for measuring this capacity [15–17]. The simplicity of the test, which requires minimal equipment and can be conducted in various settings, enhances its accessibility for sedentary individuals who used to walk in their daily life rather than running or cycling.

### Clinical parameters, gene expression, and cytokines measurements

Fasting blood samples were sent to a licensed medical laboratory to measure fasting blood sugar, HbA_1C_, total cholesterol, triglycerides, HDL, and LDL. Insulin levels were measured in serum samples using Mercodia Insulin ELISA kit (UK) according to manufacturer’s instructions. Absorbance was read using cytation5 (BioTek, imaging reader, USA). Body fat, fat-free mass, fat mass, and muscle mass were measured using TANITA body composition monitor. The handgrip tests [18–20] were performed before and after intervention. The ProcartaPlex™ Human Mix & Match cytokine multiplex kit (MAN0024966, Invitrogen) was used to simultaneously profile cytokines, including IL-1RA, IL-6, IL-8 CXCL8, MCP-1/CCL2, and TNFalpha using LUMINEX 200, according to manufacturer’s instructions. Separate standard curves are used to validate the assay for the detection and quantification of cytokines according to the manufacturer’s instructions using Xponent software. Activities of superoxide dismutase and catalase were determined using the colorimetric activity assays (EIACATC and EIASODC, respectively), according to manufacturer’s instructions (ThermoFisher Scientific, Fredrick, MD, USA). Absorbance was read using cytation5 (BioTek, imaging reader, USA). To determine the expression of *CNDP2* and three myosin heavy chain (*MYH*) genes in m. gastrocnemius, RNA sequencing was used, as previously described [9]. In brief, RNA was extracted from tissue samples of 791 individuals from the GTEx project, followed by library preparation and sequencing using an Illumina platform. The resulting raw data underwent quality control, including trimming of adapters and filtering of low-quality reads. Clean reads were then aligned to the reference genome, and gene expression levels were quantified using bioinformatics tools to obtain normalized expression values, facilitating the analysis of CNDP2 expression across different tissues. Expression of the *CNDP2* gene was presented in transcripts per kilobase million (TPM). The expression of myosin heavy chain genes (*MYH1, MYH2*, and *MYH7*) was used to determine muscle fiber composition.

### Evaluation of muscle fiber composition

Muscle fiber composition of m. gastrocnemius (GTEx cohort) was estimated in the 791 individuals based on the expression of the myosin heavy chain 1 (*MYH1*; determining fast glycolytic phenotype, i.e., type IIX muscle fibers), myosin heavy chain 2 (*MYH2*; determining fast oxidative phenotype, i.e., type IIA muscle fibers), and myosin heavy chain 7 (*MYH7*; determining slow phenotype, i.e., type I muscle fibers) genes. Given that the TPM count of each gene is proportional to the amount of each fiber type, to estimate muscle fiber type proportions, the expression (TPM) of each of the three genes (*MYH1, MYH2*, and *MYH7*) was divided by the sum of the expression of the three genes [21].

### Metabolomics and statistics

Established protocols were used for untargeted metabolomics of serum samples from all participants using Metabolon’s platform [22]. Metabolomics data of 1039 known and 259 unknown identities was median-scaled, and imputed for missing values using minimum values across batches from the median-scaled data. The data was then natural log transformed and unknown metabolites were excluded from the downstream statistical steps. The difference in 6-minute walking distance (Δ6-MWD) was calculated as the post-exercise score minus the baseline score (pre-exercise) for each participant. A cube-root transformation was applied to each participant’s difference score to address the potential skewness in the distribution and eliminate the influence of the outliers. This was followed by the categorization of the transformed difference scores into tertiles, which split the dataset into three groups based on the magnitude of Δ6-MWD. These groups represented low, medium, and high levels of change in physical performance as measured by the 6-MWT.

Principal component analysis (PCA) was performed to assess the quality of the data. The highest discriminant metabolites associated with the tertiles of Δ6-MWD were found using an OPLS model. Univariate analysis was conducted using linear regression taking metabolites as the response variable and tertiles as the explanatory variable while correcting for age, BMI, and training period. The p-values were adjusted using false discovery rate (FDR) correction. Functional enrichment analysis was performed on all nominally significant metabolites listed from the univariate analysis using Fisher’s exact test and p-values were adjusted by the FDR correction. The sub-pathways were previously predefined using Metabolon, and those with less than three top hits were dropped. In the validation cohort from the GTEx project, the association analysis between *CNDP2* gene expression and the percentage of slow (type I), fast oxidative (type IIA), and fast glycolytic (type IIX) muscle fibers was performed using multiple regression adjusted for covariates (age, sex). To make scatter plots, the Pearson correlation coefficient was used to reflect the linear-related degrees of two variables (*CNDP2* gene expression and the percentage of slowtwitch muscle fibers) in females and males. The p-values < 0.05 were considered statistically significant.

## RESULTS

### General characteristics of participants

Table 1 shows the characteristics of participants displayed as difference (post-exercise – pre-exercise) and categorized by tertiles of Δ6-MWD. No significant difference in the characteristics and clinical parameters was observed among the three groups. Additional details regarding the values of pre- and post-exercise measurements were displayed in supplementary table (S1).

**TABLE 1:** Demographic characteristics and clinical parameters of participants displayed as difference (post-exercise – pre-exercise) and categorized by tertiles of Δ6-MWD.

	Low aerobic capacity (n =15)	Medium aerobic capacity (n = 14)	High aerobic capacity (n = 14)	p-value
Δ 6-MWD (meters)	27 (18–52)	138 (102–158.5)	275.5 (228–364.5)	7.72 × 10^−9^
BMI	-0.2 (-0.5–0.3)	-0.15 (-0.48–0.08)	0.15 (-0.1–0.27)	0.456
Weight (kg)	-0.3 (-0.85–0.85)	-0.45 (-1.12–0.58)	0.5 (-0.03–0.9)	0.405
Body fat	0 (-0.01–0)	0 (-0.01–0.01)	0.01 (0–0.01)	0.348
Fat free mass (kg)	-0.2 (-0.55–0.55)	0.15 (-0.18–0.68)	-0.05 (-0.92–0.45)	0.869
Fat mass (kg)	0.2 (-0.6–0.7)	0 (-0.6–0.6)	0.4 (-1.35–0.92)	0.937
Muscle mass (kg)	-0.2 (-0.55–0.55)	0.15 (-0.18–0.6)	-0.05 (-0.83–0.45)	0.872
MET	435 (41–543.25)	884 (153.75–1687.12)	871.5(-262–1343)	0.213
Handgrip L	1.2 (-0.1–2.6)	2.05 (1.72–3.32)	2.95 (-0.38–3.78)	0.490
Handgrip R	1 (-0.75–2.2)	3.1 (1.8–4.6)	1.9 (0.12–3.6)	0.068
Insulin (mU/L)	0.62 (-1.75–1.51)	-0.26 (-1.41–2.27)	-1.04 (-4.63–1.72)	0.707
FBS (mmol/L)	0.1 (-0.3–0.75)	-0.05 (-0.27–0.22)	0.1 (-0.08–0.27)	0.583
Total Cholesterol (g/dl)	-2 (-18.5–13)	-1.5 (-13.5–7.75)	0 (-10–7)	0.824
Triglycerides (g/dl)	6 (-6.5–18)	6.5 (-4.75–15.5)	-1.5 (-7.5–6.25)	0.241
HDL (g/dl)	2 (-3–5.75)	-3.5 (-4.75–2)	-2.5 (-6–0)	0.350
LDL (g/dl)	0 (-19.5–8.5)	1 (-11.02–5.75)	3.5 (-3.25–10.5)	0.411
HbA1C	0.12 (-0.31–0.41)	0.19 (-0.06–0.53)	0.12 (0.04–0.31)	0.853
Total cholesterol HDL ratio	0 (-0.52–0.18)	0 (-0.1–0.2)	0.15 (0–0.2)	0.271
SOD (u/ml)	0.01 (-0.14–0.38)	0.18 (-0.07–0.39)	0.07 (-0.18–0.23)	0.628
Catalase (u/ml)	0.35 (0.02–0.86)	0.21 (-0.31–0.77)	0.06 (0.01–0.44)	0.820
IL 6 (pg/ml)	0 (-22.94–0)	0 (0–9.85)	0 (-2.96–5.74)	0.097
IL 8 CXCL8 (pg/ml)	1.18 (0–4.94)	0.08 (-1.37–1.54)	0.58 (-0.69–2.59)	0.824
IL 1RA (pg/ml)	2.48 (-310.5–247.9)	-2.61 (-116.3–374.4)	22.43 (-29.08–418.7)	0.915
TNF alpha (pg/ml)	0 (0–3.52)	0 (-1.76–3.52)	0 (0–3.52)	0.850
MCP 1 CCL2 (pg/ml)	84.4 (-2.74–174.15)	20.4 (-18.7–87.72)	67.68 (-0.25–259.93)	0.526

Transformed difference scores were divided into tertiles based on the magnitude of Δ6-MWD. These groups represented low, medium, and high levels of change in physical performance as measured by the 6-MWT. Data are presented as the median (IQR) of the difference between the post-exercise and the baseline for each clinical measurement. The differences between the tertiles were analysed using Kruskal-Wallis test and p-value < 0.05 was considered statistically significant.

### Multivariate analysis

**FIG. 1:**
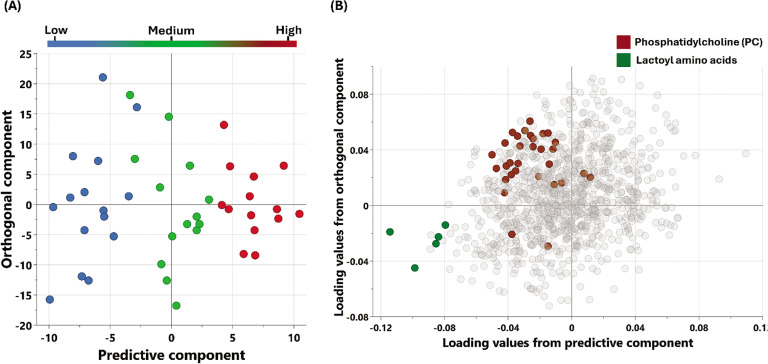
Multivariate OPLS-DA Model associated with metabolomic changes in physical performance (Δ6-MWD): (A) OPLS-DA scores plot: Separation of individuals based on global metabolomic patterns associated with changes in physical performance assessed using (Δ6-MWD). The x-axis represents the predictive component of the model (variability explained by the metabolite profile), while the y-axis represents the orthogonal component (variation not directly related to the outcome). The model explains 89% of the variation in physical performance (R2Y = 0.89), with a modest predictive ability (Q2 = 0.078). (B) Loadings Plot: This plot identifies the metabolites contributing to the separation observed in (A). Enriched pathways associated with increased 6-minute walking distance (Δ6-MWD) are highlighted, reflecting key metabolic shifts contributing to enhanced physical performance.

The metabolic signatures of the participants were analyzed using non-targeted metabolomics. OPLS-DA (Figure 1) was utilized to identify the best distinguishing components among the three studied groups.

### Univariate analysis

**FIG. 2:**
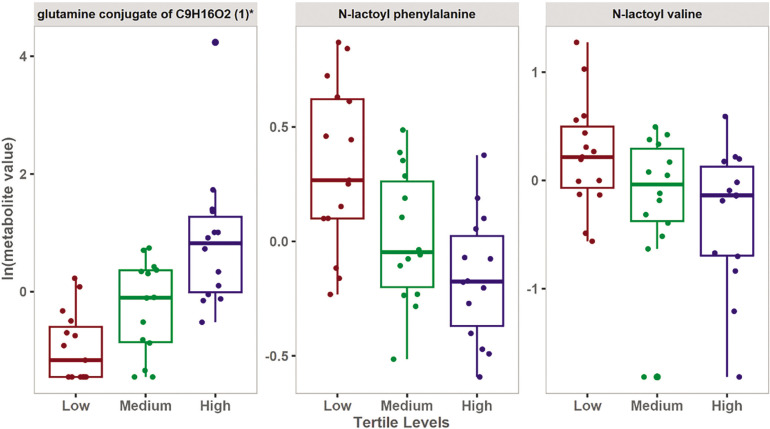
Boxplots showing the top FDR significant metabolites associated with the levels of change in 6-MWD.

Univariate analysis included paired Student’s t-test and fold change analysis to detect changes in metabolite levels among the three groups. Seventy-three metabolites were statistically significant at a nominal p-value of ≤ 0.05, however after correcting for multiple comparisons using false discovery rate (FDR), only 3 metabolites remained significant (Figure 2). Table 2 shows the top significant metabolites differentiating the three groups. Supplementary table (S2) shows all the significant 73 metabolites. The same analysis was repeated, but considering the independent variable (Δ6-MWD) as a continuous variable rather than converting into a nominal scale. The results of this analysis are shown in the supplementary table (S3).

**TABLE 2:** Results from the linear regression analysis, correcting for age, BMI, and training period.

Metabolites	Super-pathway	Sub-pathway	Estimate	SE	p-value	FDR
N-lactoyl phenylalanine	Amino Acid	Lactoyl Amino Acid	-0.314	0.063	1.54 × 10^−5^	0.016

Glutamine conjugate of C9H16O2	Partially Characterized Molecules	Partially Characterized Molecules	0.731	0.159	4.97 × 10^−5^	0.026

N-lactoyl valine	Amino Acid	Lactoyl Amino Acid	-0.448	0.107	1.65 × 10^−4^	0.05

N-lactoyl leucine	Amino Acid	Lactoyl Amino Acid	-0.277	0.076	7.74 × 10^−4^	0.17

Pyruvate	Carbohydrate	Glycolysis, Gluconeogenesis, and Pyruvate Metabolism	-0.267	0.073	8.21 × 10^−4^	0.17

Gamma-glutamyl glutamate	Peptide	Gamma-glutamyl Amino Acid	0.302	0.086	1.24 × 10^−3^	0.202

N-lactoyl tyrosine	Amino Acid	Lactoyl Amino Acid	-0.378	0.11	1.43 × 10^−3^	0.202

N-lactoyl isoleucine	Amino Acid	Lactoyl Amino Acid	-0.258	0.076	1.56 × 10^−3^	0.202

### Functional enrichment analysis

**FIG. 3:**
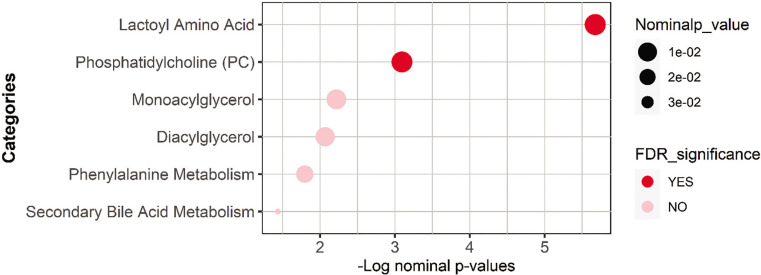
Bubble plot showing the enrichment analysis performed using Fisher’s exact test on the nominally significant metabolites.

Functional enrichment analysis was performed on nominally significant metabolites list from the univariate analysis using Fisher’s exact test and was followed by the FDR multiple testing correction method. The sub-pathways were previously predefined using Metabolon’s software, and those with less than three top hits were dropped. Results are presented in Table 3 and Figure 3.

**TABLE 3:** Results from the functional enrichment performed on all nominally significant metabolites from the linear regression analysis using Fisher’s exact test.

Sub-pathways	p-value	FDR
Lactoyl Amino Acid	0.000	0.000
Phosphatidylcholine (PC)	0.001	0.042
Monoacylglycerol	0.006	0.212
Diacylglycerol	0.009	0.224
Phenylalanine Metabolism	0.016	0.336
Secondary Bile Acid Metabolism	0.037	0.641

### CDNP2 gene expression analysis

**FIG. 4:**
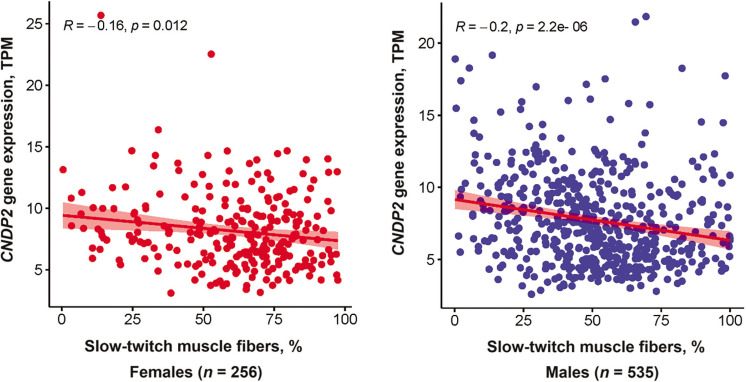
Negative correlation between *CNDP2* gene expression and the percentage of slow-twitch muscle fibres in 791 individuals from the GTEx project.

*CNDP2* gene expression was negatively associated with the percentage of slow-twitch (type I) muscle fibers (*p* < 0.0001, adjusted for age and sex). This association remained significant when the analysis was performed separately for females and males (Figure 4). On the other hand, *CNDP2* gene expression was positively associated with fast glycolytic (type IIX) muscle fibers (*p* < 0.0001, adjusted for age and sex). No association between *CNDP2* gene expression and fast oxidative (type IIA) muscle fibers was found.

## DISCUSSION

The aim of this study was to investigate potential associations between physical performance levels and metabolic parameters by comparing the metabolic profiles of participants categorized according to their difference in the 6-minute walking distance into low, medium, and high. We demonstrated that a low response to exercise training, evidenced by minimal improvement in the 6-minute walking distance, has been associated with elevated levels of N-lactoyl amino acids.

The 6-minute walking test is a valuable tool for evaluating functional capacity and fitness. The test provides insights into various bodily systems during exercise, including the pulmonary and cardiovascular systems, blood circulation and metabolism [23]. The difference in 6-minute walking distance (Δ6-MWD) between pre- and post-training provides insight into the person’s exercise response.

The results of the multivariate analysis showed that low Δ6-MWD was associated with an increase in metabolites belonging to N-lactoyl amino acids. Moreover, univariate analysis showed that N-lactoyl phenylalanine (Lac-Phe) and N-lactoyl valine have a significant inverse relationship with Δ6-MWD. These results were further validated by the enrichment analysis which showed a significant association between N-lactoyl amino acids pathway and the Δ6-MWD.

N-lactoyl-amino acids represent a new and uncharacterized class of mammalian metabolites which are found in many tissues and can approach micromolar concentrations in human plasma. N-lactoylamino acids are synthesized by the cytosolic non-specific dipeptidase (CNDP2) also called carnosine dipeptidase-2 through a process called reverse proteolysis [24].

N-lactoyl amino acids have been recently associated with various physiological and pathological conditions. Yet, data in the literature about these metabolites is very scarce.

Two studies reported a marked increase in all measured N-lactoyl amino acids in obese type 2 diabetes participants compared to obese non-diabetics [25], and in diabetic retinopathy patients compared to diabetics without retinopathy [26]. Additionally, Sharma et al. [27] revealed that N-lactoyl-amino acids levels were significantly increased in patients with mitochondrial encephalomyopathy lactic acidosis and stroke-like episodes compared with controls, suggesting an important involvement of these metabolites in mitochondrial disorders. Relatedly, N-lactoyl phenylalanine, the most representative of N-lactoyl amino acid, was shown to be associated with mitochondrial dysfunction [28] and overload [29].

Interestingly, N-lactoyl phenylalanine was recently demonstrated to be one of the top exercise-regulated metabolites in humans [30]. Moreover, this exercise-inducible metabolite was demonstrated to act as a blood-borne signalling metabolite to suppress feeding and obesity, and influence systemic energy balance [30].

Although systemic lactate did not differ among participants (Supplementary Figure S4), one possible explanation for the presence of higher levels of lactoyl amino acids in the low Δ6-MWD group in our study could be the increase of muscular lactate production in this group. Indeed, higher lactate levels were associated with shorter distances walked in 6-MWT [31]. Relatedly, Li-Gao et al. [32] hypothesized that some N-lactoyl amino acids may serve as a “metabolic sink” for lactate and could function to buffer the deleterious effects of lactate on glucose homeostasis.

Another plausible explanation for higher levels of lactoyl amino acids in the low Δ6-MWD group is the elevated levels of phenylalanine and branched-chain amino acids (BCAA) in this group. In fact, multiple studies have demonstrated that elevated phenylalanine and BCAA levels are strongly linked to impaired cardiac function, heart failure, and increased cardiovascular mortality [33, 34]. Relatedly, Jansen et al. [24] showed that N-lactoyl phenylalanine levels were increased in patients with phenylketonuria with increased plasma phenylalanine levels, suggesting that N-lactoyl phenylalanine is a scavenging metabolite that helps regulate and remove excess phenylalanine from the blood rather having a causative effect. Nonetheless, the role of N-lactoyl amino acids in human health remains a topic of ongoing investigation. A comprehensive understanding of these novel metabolites is still lacking. Therefore, there is a pressing need to further elucidate their role in human health and disease.

One reason for poor tolerance to aerobic exercise may be a low percentage of slow-twitch muscle fibers [35]. We, therefore, hypothesized that a low percentage of slow-twitch muscle fibers would be associated with increased expression of the *CNDP2* gene, which codes for an enzyme responsible for Lac-Phe synthesis. To validate our findings from the metabolomics study, we tested the association between *CNDP2* gene expression and muscle fiber composition in the GTEx cohort. Interestingly, the results showed that *CNDP2* gene expression was negatively associated with slow-twitch muscle fibers (*p* < 0.0001) and positively associated with fast glycolytic muscle fibers (*p* < 0.0001). The significant association between *CNDP2* gene expression and the type of muscle fibers in the validation cohort further supports the role of N-lactoyl amino acids in exercise physiology.

Indeed, slow-twitch fibers are more efficient at oxidizing lactate, while fast-twitch glycolytic fibers produce and accumulate lactate more rapidly. Individuals with a higher proportion of fast-twitch glycolytic fibers may not tolerate long distances due to their reliance on anaerobic metabolism. This can cause muscle fatigue, cramps, and a decrease in exercise performance [36]. Concordantly, Guilherme et al. [37] reported that the *CNDP2* rs6566810 AA genotype (which predicts low expression of *CNDP2* in skeletal muscle [9]) is overrepresented in international-level Brazilian endurance athletes. This suggests that the genetic predisposition could be, at least in part, a strong determinant of athletic performance, and that Lac-Phe could serve as a biomarker of this genetic background. Further research is necessary to fully understand the role of *CNDP2* and N-lactoyl amino acids in athletic performance.

Our results showed also a significant difference in glutamine conjugate of C_9_H_16_O_2_ between the 3 groups. Glutamine plays a crucial role in various physiological processes, including energy production and glutathione synthesis. Interestingly, glutamine has been shown to have an anti-fatigue function, and its role has been widely investigated in sports nutrition [38]. Relatedly, glutamine supplementation was shown to benefit athletes by enhancing strength, performance, recovery, and immune function [39]. Moreover, glutamine metabolism is upregulated in cardiomyocytes under oxidative stress to maintain ATP and glutathione levels, thereby exerting a cardioprotective effect [40]. The increased levels of the glutamine conjugate in the high-response group could suggest enhanced glutamine synthesis, which may contribute, at least partially, to the improved response in this group. However, glutamine conjugates are partially characterized molecules, and further research is needed to fully elucidate the role of these metabolites.

While the 6-MWT is effective for monitoring changes in aerobic capacity over time in both healthy beginners and sedentary individuals with limited exercise experience or technique, it is important to recognize that our study evaluated exercise response solely based on changes in 6-minute walking distance, which is a noted limitation. Additionally, although all participants were encouraged to maintain a balanced diet, they did not adhere to a specific, predefined dietary regimen, and this could be considered a limitation of this study. Furthermore, the validation cohort exhibited a significantly different age range compared to the exercise intervention group, which may contribute to the limitations of this study. The study also did not address the potential role of pulmonary and cardiovascular responses in the variability of exercise tolerance, which could also be important factors alongside the metabolic markers studied highlighting additional limitation in this study.

## CONCLUSIONS

In this study, a low response to exercise training, as indicated by limited improvement in the 6-minute walking distance, has been linked to elevated levels of N-lactoyl amino acids. This association suggests a potential role for N-lactoyl amino acids in influencing exercise performance and adaptation. We hypothesized that low levels of Lac-Phe, a specific N-lactoyl amino acid, may be indicative of a metabolic state that favors enhanced aerobic capacity. This raises the intriguing question of whether these N-lactoyl amino acids merely serve as biomarkers reflecting heightened levels of lactate, phenylalanine, and BCAA, or if they play a more active role as protective agents, working to counterbalance and eliminate the surplus of these molecules. Further investigation into the mechanisms underlying this relationship could provide valuable insights into optimizing exercise training outcomes and tailoring interventions for individuals with varying metabolic profiles. By understanding how N-lactoyl amino acids impact aerobic capacity, we may uncover novel strategies to improve exercise responsiveness and overall fitness levels. The identification of N-lactoyl amino acids as metabolic biomarkers opens up numerous avenues for research and practical applications in sports science. By leveraging these findings, future studies can significantly enhance our understanding of exercise physiology and improve interventions aimed at maximizing exercise tolerance across diverse populations.

## Supplementary Material

N-Lactoyl amino acids as metabolic biomarkers differentiating low and high exercise response

## Data Availability

Data is available from the corresponding author upon reasonable request.
