# Supplementary material for: N-Lactoyl amino acids as metabolic biomarkers differentiating low and high exercise response
Source: Biol Sport. 2024 Dec 19;42(2):331–44. doi: 10.5114/biolsport.2025.145912 (PMC11963115; doi:10.5114/biolsport.2025.145912)
Supplement: N-Lactoyl amino acids as metabolic biomarkers differentiating low and high exercise response [file JBS-42-55337-s1.pdf]

APPENDIX

SUPPLEMENTARY TABLE (S1). Demographic characteristics of participants categorized by tertiles of 6-minute walking test and before and after training.

Variable	Tertile 1 (n = 15)			Tertile 2 (n = 14)			Tertile 3 (n = 14)			P*
**Height m**	**1.59 (0.04)**			**1.59 (0.06)**			**1.6 (0.08)**			**0.999**
**Age**	**22 (19.5–22.5)**			**20.5 (20–25.25)**			**21 (20.25–22)**			**0.995**
	**Before**	**After**	**p**	**Before**	**After**	**p**	**Before**	**After**	**p**	
6WT Distance m	563.6 (45.58)	601.07 (45.87)	0.000	585 (86.15)	719.57 (78.54)	0.000	535.07 (81.74)	858.6 (156.77)	0.000	0.204
BMI	23.88 (5.35)	23.81 (5.17)	0.553	27.5 (5.69)	27.26 (5.58)	0.113	25.52 (6.01)	25.43 (5.37)	0.664	0.241
Weight Kg	60.19 (14.04)	60.21 (13.6)	0.962	69.56 (15.07)	69.1 (14.76)	0.261	65.63 (17.65)	65.65 (16.36)	0.964	0.279
Body fat	0.29 (0.1)	0.28 (0.1)	0.369	0.36 (0.09)	0.36 (0.08)	0.500	0.31 (0.09)	0.31 (0.09)	0.908	0.105
Fat free mass Kg	41.83 (3.91)	41.89 (3.55)	0.754	45.65 (3.86)	45.68 (3.57)	0.921	44.34 (5.38)	44.4 (4.54)	0.881	0.073
Fat mass Kg	17.71 (10.31)	18.38 (10.28)	0.353	27.44 (10.19)	28.44 (12.45)	0.503	21.81 (13.27)	21.61 (12.74)	0.659	0.080
muscle mass Kg	39.68 (3.72)	39.75 (3.38)	0.724	43.32 (3.68)	43.36 (3.39)	0.898	42.07 (5.11)	42.15 (4.32)	0.845	0.072
MET	1286.33 (1355.61)	1295.5 (570.12)	0.979	1520.29 (860.71)	2549.96 (1361.78)	0.017	1676.57 (764.18)	2839.68 (2358)	0.087	0.597
Handgrip L	20.83 (4.6)	22.21 (5.29)	0.028	23.69 (5.69)	26.17 (6.22)	0.017	23.28 (4.28)	26.49 (5.65)	0.052	0.243
Handgrip R	22.37 (5.82)	23.09 (5.39)	0.411	24.54 (5.58)	28.86 (7.86)	0.017	25.79 (3.5)	29.87 (5.79)	0.038	0.199
Insulin mU L	15.73 (7.66)	14.37 (6.48)	0.338	13.87 (8.1)	13.21 (6.43)	0.532	12.36 (5.05)	10.21 (2.1)	0.124	0.446
FBS mmol L	5.14 (0.4)	5.33 (0.57)	0.231	5.09 (0.35)	5.11 (0.44)	0.832	4.84 (0.26)	4.94 (0.23)	0.200	0.058
HOMA IR	3.67 (1.98)	3.2 (1.5)	0.200	3.19 (1.98)	3.03 (1.51)	0.561	2.92 (1.38)	2.18 (0.44)	0.057	0.537
Total Cholesterol g dl	195.2 (32.43)	191.27 (36.53)	0.468	176.74 (31.26)	174.71 (26)	0.574	173 (19.98)	174.57 (21.34)	0.743	0.092
Triglycerides g dl	78.07 (42.27)	85.8 (59.31)	0.257	64.93 (16.51)	71.07 (23.55)	0.212	68.57 (32.73)	63.57 (22.58)	0.280	0.536
HDL g dl	60.93 (17.21)	60.6 (16.8)	0.641	64.16 (12.29)	62.14 (11.63)	0.341	61.71 (11.19)	59.29 (11.48)	0.200	0.812
LDL g dl	120 (33.97)	113.67 (35.89)	0.331	99.62 (29.2)	99.07 (26.62)	0.861	97.5 (19.73)	103.43 (15.79)	0.115	0.073
HbA1C	5.18 (0.55)	5.24 (0.3)	0.520	5.04 (0.34)	5.16 (0.46)	0.242	5.03 (0.22)	5.29 (0.35)	0.116	0.632
Total cholesterol HDL ratio	3.37 (1.09)	3.38 (1.16)	0.214	2.81 (0.59)	2.91 (0.65)	0.202	2.89 (0.57)	3.03 (0.48)	0.073	0.138
SOD u ml	1.22 (0.56)	1.45 (1.03)	0.222	0.97 (0.63)	1.03 (0.17)	0.724	1.02 (0.4)	1.13 (0.32)	0.396	0.422
Catalase u ml	19.72 (19.07–20.17)	20.07 (19.74–20.23)	0.148	19.59 (19.08–20.1)	19.99 (19.63–20.2)	0.346	20.16 (19.85–20.25)	20.25 (20.11–20.32)	0.187	0.091
IL 8 CXCL8 pg ml	2.94 (1.12–2.94)	4.48 (3.6) (4 missing)	0.771	1.12 (0.96–3.15)	2.19 (2.52) (8 missing)	NA	1.12 (0.49–2.25)	2.55 (2.53)	0.202	0.412
IL 10 pg ml	0.63 (0.42–3.23)	0.71 (0.81) (11 missing)	0.356	0.08 (0.08–0.08)	0.26 (0.32) (11 missing)	NA	0.08 (0)	0.35 (0.39) (12 missing)	NA	0.643
IL 1RA pg ml	736.01 (833.82)	686.89 (720.13)	0.838	241.53 (105.25–434.66)	438.31 (378.01)	0.297	512.7 (562.38)	604.77 (633.35)	0.673	0.288
TNF alpha pg ml	0.43 (0.43–3.95)	3.95 (0.43–3.95)	0.719	0.43 (0.43–3.95)	3.95 (0.43–3.95)	0.983	0.43 (0.43–3.95)	3.95 (0.43–3.95)	0.171	0.532
MCP 1 CCL2 pg ml	326.62 (230.19)	448.15 (266.66)	0.054	311.96 (168.87)	349.54 (202.93)	0.233	279.5 (188.89)	435.95 (324.58)	0.036	0.812

Data are presented as mean ± SD/ median (IQR) for parametric/non-parametric variables. Baseline clinical measurements between the tertiles were compared using ANOVA/Kruskal Wallis test based on the normality status of the variables (denoted by P*). Before and after measurements in each tertile were compared using Paired Students' t/Wilcoxon matched pairs test (p).

SUPPLEMENTARY TABLE (S2). Results from the linear regression analysis, correcting for age, BMI, and training period.

Metabolites	Super-pathway	Sub-pathway	Estimate	SE	p-value	FDR
N-lactoyl phenylalanine	Amino Acid	Lactoyl Amino Acid	-0.314	0.063	1.54×10^{-5}	0.016
glutamine conjugate of C9H16O2 (1)*	Partially Characterized Molecules	Partially Characterized Molecules	0.731	0.159	4.97×10^{-5}	0.026
N-lactoyl valine	Amino Acid	Lactoyl Amino Acid	-0.448	0.107	1.65×10^{-4}	0.05
N-lactoyl leucine	Amino Acid	Lactoyl Amino Acid	-0.277	0.076	7.74×10^{-4}	0.170
pyruvate	Carbohydrate	Glycolysis, Gluconeogenesis, and Pyruvate Metabolism	-0.267	0.073	8.21×10^{-4}	0.170
gamma-glutamylglutamate	Peptide	Gamma-glutamyl Amino Acid	0.302	0.086	1.24×10^{-3}	0.202
N-lactoyl tyrosine	Amino Acid	Lactoyl Amino Acid	-0.378	0.110	1.43×10^{-3}	0.202
N-lactoyl isoleucine	Amino Acid	Lactoyl Amino Acid	-0.258	0.076	1.56×10^{-3}	0.202
glycoursodeoxycholic acid sulfate (1)	Lipid	Secondary Bile Acid Metabolism	-0.818	0.259	3.11×10^{-3}	0.358
2-oxoarginine*	Amino Acid	Urea cycle; Arginine and Proline Metabolism	-0.281	0.093	4.58×10^{-3}	0.425
1-linolenoyl-GPC (18:3)*	Lipid	Lysophospholipid	-0.242	0.081	4.86×10^{-3}	0.425
N-acetyl-2-aminoadipate	Amino Acid	Lysine Metabolism	-0.329	0.110	4.92×10^{-3}	0.425
tetradecadienedioate (C14:2-DC)*	Lipid	Fatty Acid, Dicarboxylate	0.329	0.117	7.87×10^{-3}	0.627
1-stearoyl-2-dihomo-linolenoyl-GPC (18:0/20:3n3 or 6)*	Lipid	Phosphatidylcholine (PC)	-0.230	0.086	1.11×10^{-2}	0.668
3-formylindole	Xenobiotics	Food Component/Plant	-0.169	0.064	1.17×10^{-2}	0.668
isobutyrylglycine (C4)	Amino Acid	Leucine, Isoleucine and Valine Metabolism	-0.198	0.075	1.25×10^{-2}	0.668
1-oleoylglycerol (18:1)	Lipid	Monoacylglycerol	-0.216	0.082	1.25×10^{-2}	0.668
xanthurenate	Amino Acid	Tryptophan Metabolism	-0.418	0.160	1.30×10^{-2}	0.668
glucose	Carbohydrate	Glycolysis, Gluconeogenesis, and Pyruvate Metabolism	-0.044	0.017	1.33×10^{-2}	0.668
2-methylcitrate/homocitrate	Energy	TCA Cycle	-0.090	0.035	1.36×10^{-2}	0.668
1-dihomo-linolenoyl-GPC (20:3n3 or 6)*	Lipid	Lysophospholipid	-0.188	0.073	1.42×10^{-2}	0.668
phenylpyruvate	Amino Acid	Phenylalanine Metabolism	-0.150	0.059	1.51×10^{-2}	0.668
lignoceroyl sphingomyelin (d18:1/24:0)	Lipid	Sphingomyelins	-0.169	0.066	1.53×10^{-2}	0.668
metformin	Xenobiotics	Drug – Metabolic	-0.183	0.072	1.58×10^{-2}	0.668
decadienedioic acid (C10:2-DC)**	Lipid	Fatty Acid, Dicarboxylate	0.299	0.119	1.63×10^{-2}	0.668
1-stearoyl-2-oleoyl-GPC (18:0/18:1)	Lipid	Phosphatidylcholine (PC)	-0.163	0.065	1.68×10^{-2}	0.668
1-myristoyl-2-arachidonoyl-GPC (14:0/20:4)*	Lipid	Phosphatidylcholine (PC)	-0.295	0.120	1.85×10^{-2}	0.675
gamma-glutamylcitrulline*	Peptide	Gamma-glutamyl Amino Acid	0.150	0.062	2.00×10^{-2}	0.675
isoursodeoxycholate	Lipid	Secondary Bile Acid Metabolism	-0.563	0.232	2.01×10^{-2}	0.675
2-arachidonoylglycerol (20:4)	Lipid	Monoacylglycerol	-0.409	0.169	2.06×10^{-2}	0.675
HWESASXX*	Peptide	Polypeptide	-0.411	0.172	2.19×10^{-2}	0.675
cholesterol sulfate	Lipid	Sterol	-0.105	0.044	2.20×10^{-2}	0.675
1-palmitoyl-2-dihomo-linolenoyl-GPC (16:0/20:3n3 or 6)*	Lipid	Phosphatidylcholine (PC)	-0.182	0.076	2.20×10^{-2}	0.675
taurolithocholate 3-sulfate	Lipid	Secondary Bile Acid Metabolism	0.482	0.202	2.23×10^{-2}	0.675
glycerophosphoglycerol	Lipid	Glycerolipid Metabolism	-0.112	0.047	2.33×10^{-2}	0.675
undecenoylcarnitine (C11:1)	Lipid	Fatty Acid Metabolism (Acyl Carnitine, Monounsaturated)	0.368	0.156	2.36×10^{-2}	0.675
1-palmitoylglycerol (16:0)	Lipid	Monoacylglycerol	-0.303	0.131	2.59×10^{-2}	0.675
2-methylserine	Amino Acid	Glycine, Serine and Threonine Metabolism	0.189	0.082	2.64×10^{-2}	0.675
1-palmitoyl-2-oleoyl-GPC (16:0/18:1)	Lipid	Phosphatidylcholine (PC)	-0.099	0.043	2.65×10^{-2}	0.675
pristanate	Lipid	Fatty Acid, Branched	-0.446	0.194	2.72×10^{-2}	0.675

SUPPLEMENTARY TABLE (S2). Continue

Metabolites	Super-pathway	Sub-pathway	Estimate	SE	p-value	FDR
palmitoyl-linoleoyl-glycerol (16:0/18:2) [2]*	Lipid	Diacylglycerol	-0.422	0.186	2.89×10^{-2}	0.675
fructosyllysine	Amino Acid	Lysine Metabolism	-0.083	0.037	2.94×10^{-2}	0.675
N-behenoyl-sphingadienine (d18:2/22:0)*	Lipid	Ceramides	-0.374	0.165	2.97×10^{-2}	0.675
deoxycholic acid 12-sulfate*	Lipid	Secondary Bile Acid Metabolism	-0.482	0.213	2.98×10^{-2}	0.675
linoleoyl-linolenoyl-glycerol (18:2/18:3) [2]*	Lipid	Diacylglycerol	-0.419	0.186	3.03×10^{-2}	0.675
beta-alanine	Nucleotide	Pyrimidine Metabolism, Uracil containing	-0.172	0.076	3.04×10^{-2}	0.675
4-ethylphenyl sulfate	Xenobiotics	Benzoate Metabolism	0.372	0.167	3.16×10^{-2}	0.675
gamma-glutamylglutamine	Peptide	Gamma-glutamyl Amino Acid	0.072	0.032	3.23×10^{-2}	0.675
phenylalanine	Amino Acid	Phenylalanine Metabolism	-0.064	0.029	3.24×10^{-2}	0.675
1-linoleoylglycerol (18:2)	Lipid	Monoacylglycerol	-0.187	0.084	3.26×10^{-2}	0.675
1-palmitoyl-2-oleoyl-GPE (16:0/18:1)	Lipid	Phosphatidylethanolamine (PE)	-0.344	0.156	3.32×10^{-2}	0.675
picolinoylglycine	Lipid	Fatty Acid Metabolism (Acyl Glycine)	-0.270	0.123	3.51×10^{-2}	0.688
1-palmitoyl-2-palmitoleoyl-GPC (16:0/16:1)*	Lipid	Phosphatidylcholine (PC)	-0.158	0.073	3.66×10^{-2}	0.688
1-oleoyl-2-dihomo-linolenoyl-GPC (18:1/20:3)*	Lipid	Phosphatidylcholine (PC)	-0.128	0.060	3.82×10^{-2}	0.688
5-hydroxyindole sulfate	Amino Acid	Tryptophan Metabolism	-0.368	0.172	3.91×10^{-2}	0.688
phenyllactate (PLA)	Amino Acid	Phenylalanine Metabolism	-0.124	0.058	4.05×10^{-2}	0.688
behenoyl sphingomyelin (d18:1/22:0)*	Lipid	Sphingomyelins	-0.117	0.055	4.09×10^{-2}	0.688
ursodeoxycholate	Lipid	Secondary Bile Acid Metabolism	-0.466	0.221	4.17×10^{-2}	0.688
1-stearoyl-2-oleoyl-GPE (18:0/18:1)	Lipid	Phosphatidylethanolamine (PE)	-0.373	0.177	4.21×10^{-2}	0.688
ribitol	Carbohydrate	Pentose Metabolism	-0.074	0.035	4.27×10^{-2}	0.688
1-stearoyl-2-linoleoyl-GPC (18:0/18:2)*	Lipid	Phosphatidylcholine (PC)	-0.109	0.052	4.27×10^{-2}	0.688
oleoyl-linoleoyl-glycerol (18:1/18:2) [2]	Lipid	Diacylglycerol	-0.219	0.105	4.30×10^{-2}	0.688
picolinate	Amino Acid	Tryptophan Metabolism	-0.239	0.115	4.46×10^{-2}	0.688
4-hydroxyphenylacetylglutamine	Peptide	Acetylated Peptides	-0.279	0.134	4.48×10^{-2}	0.688
delta-CEHC	Cofactors and Vitamins	Tocopherol Metabolism	-0.360	0.174	4.54×10^{-2}	0.688
1-linoleoyl-GPA (18:2)*	Lipid	Lysophospholipid	-0.196	0.095	4.60×10^{-2}	0.688
1-stearoyl-GPC (18:0)	Lipid	Lysophospholipid	-0.112	0.054	4.61×10^{-2}	0.688
2-hydroxy-3-methylvalerate	Amino Acid	Leucine, Isoleucine and Valine Metabolism	-0.139	0.068	4.65×10^{-2}	0.688
tyramine O-sulfate	Amino Acid	Tyrosine Metabolism	-0.401	0.195	4.68×10^{-2}	0.688
myristoylcarnitine (C14)	Lipid	Fatty Acid Metabolism (Acyl Carnitine, Long Chain Saturated)	-0.105	0.051	4.71×10^{-2}	0.688
1,2-dipalmitoyl-GPC (16:0/16:0)	Lipid	Phosphatidylcholine (PC)	-0.115	0.056	4.78×10^{-2}	0.688
palmitoyl-linoleoyl-glycerol (16:0/18:2) [1]*	Lipid	Diacylglycerol	-0.376	0.183	4.78×10^{-2}	0.688
linoleoyl-arachidonoyl-glycerol (18:2/20:4) [2]*	Lipid	Diacylglycerol	-0.361	0.177	4.88×10^{-2}	0.692

SUPPLEMENTARY TABLE (S3). Results from the linear regression analysis (treating the difference in 6-minute walking difference as continuous variable), correcting for age, BMI, and training period.

Metabolites	Super-pathway	Sub-pathway	Estimate	SE	p-value	FDR
N-lactoyl phenylalanine	Amino Acid	Lactoyl Amino Acid	-0.139	0.032	0.00012	0.13
N-lactoyl leucine	Amino Acid	Lactoyl Amino Acid	-0.138	0.036	0.00053	0.15
N-lactoyl isoleucine	Amino Acid	Lactoyl Amino Acid	-0.135	0.035	0.00054	0.15
N-lactoyl valine	Amino Acid	Lactoyl Amino Acid	-0.198	0.043	0.00065	0.15
Glutamine conjugate of C9H16O2	Partially Characterized Molecules	Partially Characterized Molecules	0.287	0.054	0.00075	0.15
Phenylalanine	Amino Acid	Phenylalanine Metabolism	-0.099	0.085	0.0017	0.29
Pyruvate	Carbohydrate	Glycolysis, Gluconeogenesis, and Pyruvate Metabolism	-0.041	0.029	0.002	0.29
N-lactoyl tyrosine	Amino Acid	Lactoyl Amino Acid	-0.120	0.013	0.0033	0.43

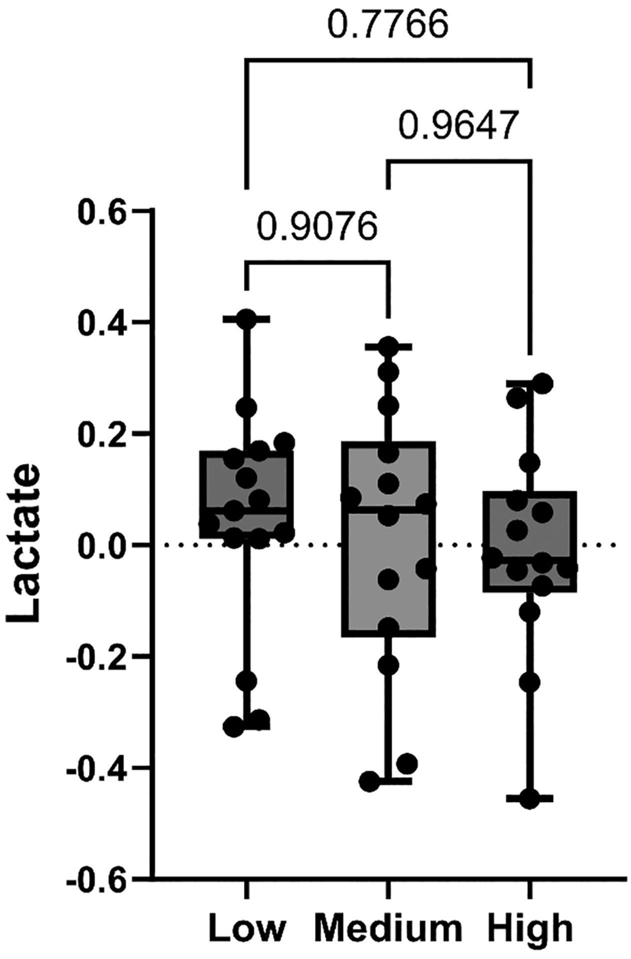

SUPPLEMENTARY FIGURE (S4): Lactate levels when compared among the three groups using ANOVA.
